# 

**Published:** 1884-07

**Authors:** 


PAGE

ORIGINAL COMMUNICATIONS.—Texas Cattle Fever. D. E. Salmon, D.V.M. .......... 213
Art and Science of Veterinary Medicine. Dr. Robert Ward, F.R.C.V.S. .......... 235
Abnormal Habits in Animals. Felix L. Oswald, M.D. .......... 241
The Massachusetts Veterinary Association. F. S. Billings, V.S. .......... 245
Comparative Physiology of Menstruation. Alfred Wiltshire, M.D., F.R.C.P. .......... 254
The Imperial Board of Health of Germany .......... 263
Excision of portions of the Intestine in the Dog .......... 273

EDITORIAL.—The Bureau of Animal Industry—Trichinæ in American Pork—The Protection of Ignorance by the U. S. Government—Tuberculosis in Cattle—Statistics in reference to the Frequency of Tuberculosis in Animals slaughtered at Augsburg in 1883—Empirics and the Proposed Veterinary Association in Missouri—Plagiarism—The American Veterinary College—Animal Castration .......... 280-294

OBITUARY.—Prof. Dr. Ludwig Franck; Edwin M. Fitzgerald, D.V.S.; Dr. George W. Bowler; Superior Medical Councillor D. V. Straub; Rudolf Zaugger .......... 295

CORRESPONDENCE .......... 297

REPORTS.—Montreal Veterinary College—Veterinary Medical Association of New Jersey .......... 301

REVIEWS.—The Influence of Heredity and Contagion on the Propagation of Tuberculosis, and the Prevention of Injurious Effects from Consumption of the Flesh and Milk of Tuberculous Animals—The Urine in Disease—Books and Pamphlets received .......... 305

